# The SUMO E3 ligase activity of ORF45 determines KSHV lytic replication

**DOI:** 10.1371/journal.ppat.1010504

**Published:** 2022-04-28

**Authors:** Zhenshan Liu, Xin Wang, Chengrong Liu, Hongying Deng, Wenshu Li, Xiaoqian Wang, Xue Xu, Maggie Z. X. Xiao, Chunxia Wang, Yucai Zhang, Joyce Fu, Fanxiu Zhu, Qiming Liang

**Affiliations:** 1 Center for Immune-Related Diseases at Shanghai Institute of Immunology, Ruijin Hospital, Shanghai Jiao Tong University School of Medicine, Shanghai, China; 2 Department of Immunology and Microbiology, Key Laboratory of Cell Differentiation and Apoptosis of Chinese Ministry of Education, Shanghai Jiao Tong University School of Medicine, Shanghai, China; 3 Department of Biological Science, Florida State University, Tallahassee, Florida, United States of America; 4 College of Arts and Sciences, New York University Shanghai, Shanghai, China; 5 Joint Ph.D. Degree Program between SJTU-SM and HUJI-MED, Shanghai Jiao Tong University, Shanghai, China; 6 Faculty of Medicine, University of Alberta, Edmonton, Alberta, Canada; 7 Department of Critical Care Medicine, Shanghai Children’s Hospital, Shanghai Jiao Tong University, Shanghai, China; 8 Institute of Pediatric Infection, Immunity and Intensive Care Medicine, Shanghai Jiao Tong University School of Medicine, Shanghai, China; 9 Department of Statistics, University of California, Riverside, Riverside, California, United States of America; 10 State Key Laboratory of Microbial Metabolism, Shanghai Jiao Tong University, Shanghai, China; 11 Research Center of Translational Medicine, Shanghai Children’s Hospital, Shanghai Jiao Tong University, Shanghai, China; Wistar Institute, UNITED STATES

## Abstract

RSK1, an essential cellular kinase for Kaposi’s sarcoma-associated herpesvirus (KSHV) replication, is highly phosphorylated and SUMOylated during KSHV lytic cycle, which determine the substrate phosphorylation and specificity of RSK1, respectively. However, the SUMO E3 ligase responsible for attaching SUMO to RSK1 has not yet been identified. By genome-wide screening, we found that KSHV ORF45 is necessary and sufficient to enhance RSK1 SUMOylation. Mechanistically, KSHV ORF45 binds to SUMOs via two classic SUMO-interacting motifs (SIMs) and functions as a SIM-dependent SUMO E3 ligase for RSK1. Mutations on these ORF45 SIMs resulted in much lower lytic gene expressions, viral DNA replication, and mature progeny virus production. Interestingly, KSHV ORF45 controls RSK1 SUMOylation and phosphorylation via two separated functional regions: SIMs and amino acid 17–90, respectively, which do not affect each other. Similar to KSHV ORF45, ORF45 of Rhesus Macaque Rhadinovirus has only one SIM and also increases RSK1 SUMOylation in a SIM-dependent manner, while other ORF45 homologues do not have this function. Our work characterized ORF45 as a novel virus encoded SUMO E3 ligase, which is required for ORF45-RSK1 axis-mediated KSHV lytic gene expression.

## Author summary

The 90-kDa ribosomal S6 kinases 1 (RSK1) is an essential cellular kinase for the lytic replication of Kaposi’s sarcoma-associated herpesvirus (KSHV). Besides the phosphorylation, RSK1 is highly SUMOylated, which is required for efficient KSHV lytic replication. Here, we found that KSHV ORF45 binds to SUMO1 and SUMO2 via two classic SUMO-interacting motifs (SIMs) and functions as a SIM-dependent SUMO E3 ligase, which is necessary and sufficient to enhance RSK1 SUMOylation upon KSHV lytic replication. Interestingly, KSHV ORF45 controls RSK1 SUMOylation and phosphorylation via two separated functional regions: SIMs and amino acid 17–90, respectively, which do not affect each other. Like KSHV ORF45, ORF45 of Rhesus Macaque Rhadinovirus also enhances RSK1 SUMOylation in a SIM-dependent manner, while ORF45 of Herpesvirus saimiri, Murine γ-herpesvirus 68, and Epstein-Barr virus cannot. Our data demonstrated that ORF45 as a novel virus encoded SUMO E3 ligase, which is essential for KSHV lytic replication and progeny virus production.

## Introduction

Kaposi’s sarcoma-associated herpesvirus (KSHV), also known as human herpesvirus-8 (HHV-8), is etiologically associated with a number of human cancers, such as Kaposi’s sarcoma (KS), primary effusion lymphoma, and multicentric Castleman’s disease [1,2]. KSHV belongs to the γ-herpesvirus subfamily, which includes Rhesus Macaque Rhadinovirus (RRV), Herpesvirus saimiri (HVS), Murine γ-herpesvirus 68 (MHV68), and Epstein-Barr virus (EBV) [1]. Like other herpesviruses, the KSHV life cycle consists of two distinct phases, namely latency and lytic replication. Latency is a dormant state during which most KSHV-encoded genes are silenced and only a portion of latent genes and viral microRNAs are expressed. Lytic replication results in the expressions of a panel of viral genes (immediate-early, early, and late genes), virion assembly, the release of mature progeny viruses, and *de novo* infection of other cells. As an intracellular parasite, KSHV utilizes its viral proteins to modulate cellular post-translational modification machineries, such as phosphorylation, ubiquitination, and SUMOylation, to escape antiviral immune surveillance and establish efficient viral replication [3], but the detailed mechanism underlying these processes still need to be further characterized.

SUMOylation is a reversible protein modification process, during which small ubiquitin-like modifiers (SUMOs) are covalently conjugated to lysine residues of the acceptor proteins. Like ubiquitination, SUMOylation requires an enzymatic cascade that involves three classes of enzymes, including the activating enzyme E1 (SAE1/UBA2), conjugating enzyme E2 (ubc9), and various E3 ligases. Sometimes, SUMO E3 ligases are not indispensable for the target modifications [4]. Humans encode three SUMO isoforms (SUMO1-3) and SUMO2 and SUMO3 are 96% identical. SUMO modification enables selected protein-protein interaction through SUMO-SIM (SUMO-interaction motif) binding, resulting in inhibition of transcriptional factors, change of protein localization, increase or decrease of enzyme activities, and alteration of protein stability [4]. We previously discovered that cellular 90-kDa ribosomal S6 kinases (RSKs), the direct substrates of ERK1/ERK2, play critical roles for KSHV lytic replication and progeny virus production [5,6]. Besides the kinase activity, SUMOylation of RSK1 primarily at K110, K335, and K421 is required for efficient KSHV lytic replication by selectively mediating kinase-substrate association, which enables the efficient binding and phosphorylation of the substrate eIF4B by RSK1 via SUMO-SIM interaction [7]. KSHV lytic replication promotes RSK1 SUMOylation, but the SUMO E3 ligase in this process remains elusive.

Open reading frame 45 (ORF45) of KSHV is an immediate early gene and a tegument structural gene [8]. KSHV ORF45 targets various host and viral factors to modulate viral replication [5,6], virion trafficking and assembly [9], and immune evasion [10,11]. KSHV ORF45 binds to host RSKs and strongly activates them by forming high molecular mass protein complexes with ERK, preventing RSKs from dephosphorylation [6,12]. ORF45-RSK axis promotes translation of a subset of viral and cellular genes during KSHV lytic replication by activating the regulatory translation initiation factor eIF4B [13]. Here, we showed that KSHV ORF45 binds to SUMO1/2 via two classic SIMs and acts as a SIM-dependent SUMO E3 ligase for RSK1, which is conserved in RRV ORF45. Mutations on the SIMs of ORF45 significantly decrease KSHV lytic replication and progeny virus production. Our finding revealed a molecular mechanism by which KSHV ORF45 controls both phosphorylation and SUMOylation of RSK1 via two separated functional regions, highlighting the critical role of ORF45 in KSHV lytic replication.

## Results

### KSHV ORF45 promotes RSK1 SUMOylation

**Fig 1:**
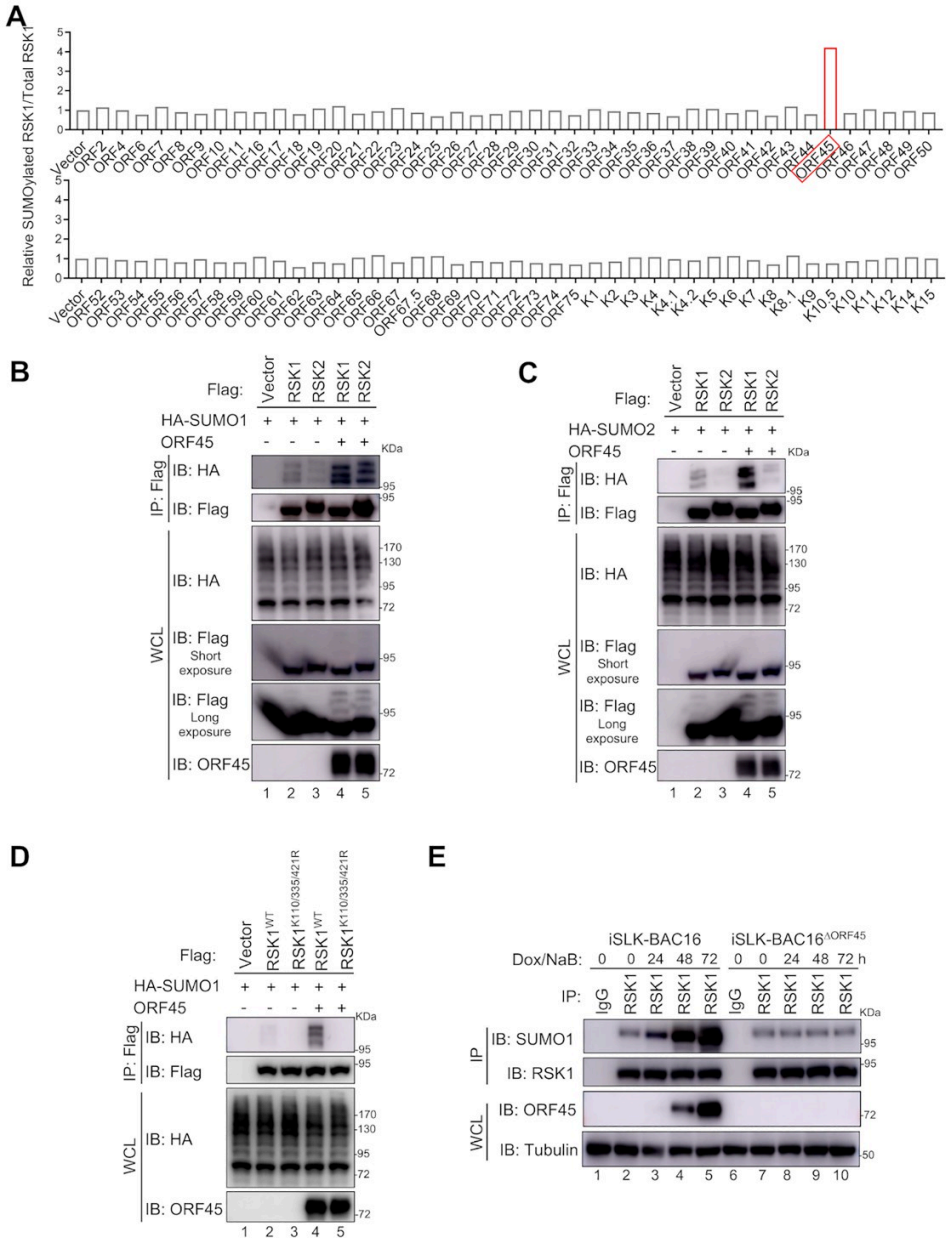
KSHV ORF45 facilitates RSK1 SUMOylation. (**A**) KSHV ORF45 enhances RSK1 SUMOylation. Individual KSHV encoded gene or vector control was co-expressed with HA-RSK1 and denatured immunoprecipitation (IP) and immunoblot (IB) were performed at 48 h post-transfection. The band intensities of SUMOylated RSK1 and total RSK1 were quantified and the ratio was calculated. (**B-C**) ORF45 promotes the SUMOylation of RSK1 and RSK2 by SUMO1/2. HEK293T cells were transfected with indicated plasmids and cell lysates were subjected to denatured IP and IB with indicated antibodies at 48 h post-transfection. (**D**) ORF45 facilitates RSK1 SUMOylation on K110, K335, and K421. (**E**) ORF45 is required for KSHV-mediated RSK1 SUMOylation. iSLK-BAC16 and iSLK-BAC16^ΔORF45^ cells were treated with doxycycline and sodium butyrate to induce KSHV lytic replication. Cells were collected at 0, 24, 48, and 72 h post-treatment and subjected to denatured IP and IB with indicated antibodies.

To determine how RSK1 is SUMOylated during KSHV lytic replication, we posited whether certain KSHV encoded viral components control RSK1 SUMOylation to benefit the viral life cycle. We performed a genome-wide screening with KSHV encoded genes to determine which viral protein is responsible for RSK1 SUMOylation. As shown in Fig 1A, only ORF45 expression dramatically enhanced RSK1 SUMOylation, while other viral proteins had little or no effect (Fig 1A and S1 Fig). Similar to RSK1, modification of RSK2 by SUMO1 or SUMO2 was also significantly increased by ORF45 (Fig 1B and 1C). In addition, ORF45 promoted the endogenous SUMOylation of RSK1 (S2A Fig) and mutations on K110/335/421 of RSK1 abolished ORF45-mediated SUMOylation (Fig 1D). Importantly, wild-type KSHV lytic replication triggered RSK1 SUMOylation, while KSHV^ΔORF45^ mutant virus did not (Fig 1E, compare lane 9 with lane 4, or lane 10 with lane 5), suggesting that ORF45 is necessary and sufficient to induce RSK1 SUMOylation during KSHV lytic replication. Since previous studies suggested that KSHV K8 has SUMO E3 ligase activity, we subsequently examined whether K8 also promotes RSK1 SUMOylation. Unlike ORF45, K8 had no effects on RSK1 SUMOylation (S2B Fig). These results indicated that ORF45 is necessary and sufficient to promote RSK SUMOylation during KSHV lytic replication.

### KSHV ORF45 interacts with SUMOs via two SIMs

**Fig 2:**
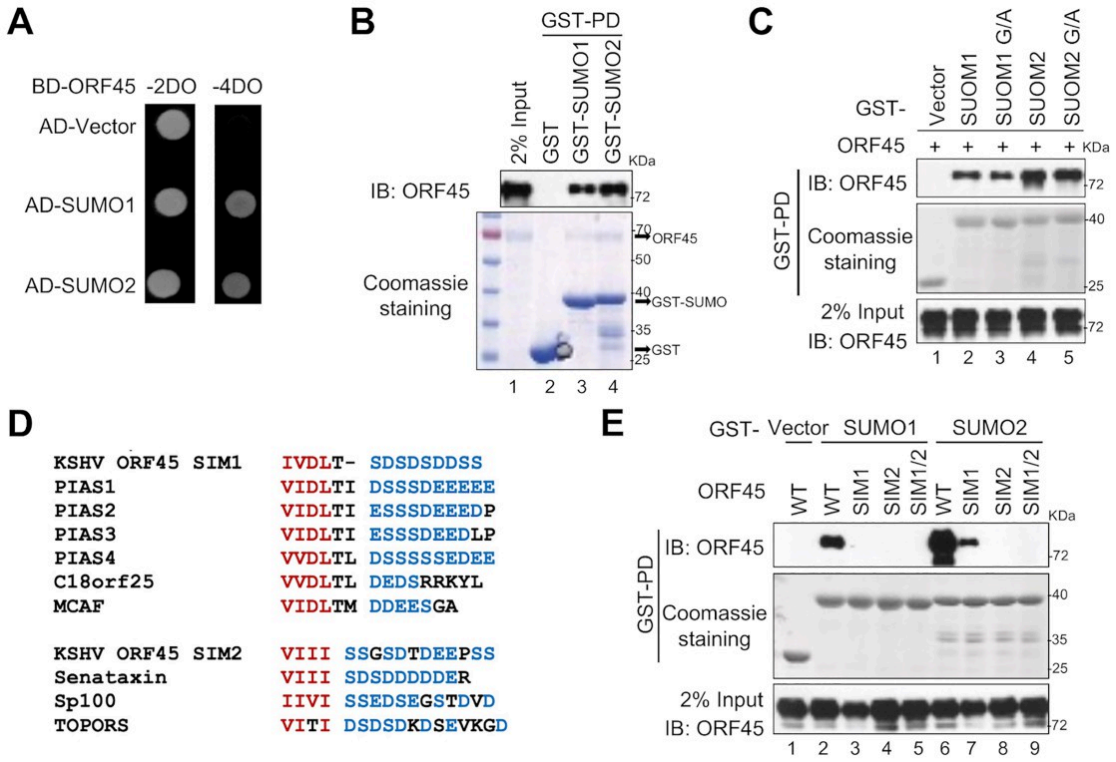
ORF45 binds to SUMO via two SIMs. (**A**) KSHV ORF45 binds to SUMO1 and SUMO2 in yeast. Yeast competent cells were co-transformed with indicated plasmids (bait plus prey). Activation of the *HIS3* and *ADE2* reporters was assessed by growth on -4DO media plates. (**B**) Recombinant KSHV ORF45 directly binds to SUMO1 and SUMO2 *in vitro*. 0.5 μg recombinant ORF45 were mixed with GST only, GST-SUMO1, or GST-SUMO2 *in vitro* for 30 min. The mixture was subjected to GST pull-down and co-precipitated proteins were determined by coomassie blue staining and IB with anti-ORF45 antibody. (**C**) KSHV ORF45 non-covalently binds to SUMO1/2. (**D**) KSHV ORF45 contains two SIMs. Alignment between ORF45 SIM1/2 and well-characterized SIMs from indicated genes. Red: core sequence; Blue: acidic amino acid tail. (**E**) Mutations of SIMs affect ORF45-SUMOs interaction. GST only, GST-SUMO1, or GST-SUMO2 were mixed with the cell lysates from HEK293T transfected with ORF45, ORF45^SIM1^, ORF45^SIM2^, or ORF45^SIM1/2^ for 30 min and the mixture was subjected to GST pull-down assay.

Next, we investigated the underlying mechanisms by which KSHV ORF45 facilitates RSK1 SUMOylation. From our yeast two-hybrid screening, we found that full-length ORF45 interacted with SUMO2. We further confirmed that ORF45 interacted with both SUMO1 and SUMO2 in yeast (Fig 2A). The direct interaction between KSHV ORF45 and SUMO1/2 was confirmed by GST pull-down assay in a cell-free system. As shown in Fig 2B, when glutathione-Sepharose beads conjugated with GST-SUMO1, GST-SUMO2 or GST-only proteins were incubated with purified His-tagged KSHV ORF45 protein, ORF45 was pulled down by both GST-SUMO1 and GST-SUMO2, but not by control GST-only beads (Fig 2B). Next, we asked whether ORF45 interacts with SUMO covalently or noncovalently. We mutated the C-terminal glycine to alanine to generate SUMO G/A mutants (SUMO1^G96A/G97A^ and SUMO2^G92A/G93A^), which cannot mature to modify an acceptor lysine on the target protein, and used these mutants to perform *in vitro* GST pull-down assay. The results suggested that KSHV ORF45 directly and non-covalently interacts with both SUMO1 and SUMO2 (Fig 2C).

To map the SUMO-interacting domains of KSHV ORF45, we initially performed a sequence alignment analysis and noticed that the amino acids _237_IVDL_240_ (SIM1) matched the core sequence of the SIM found in PIAS family, c18orf25 and MCAF, while the amino acids _328_VIII_331_ (SIM2) matched the core sequence of the SIM found in Senataxin, Sp100, and Topors (Fig 2D). To exam the functional importance of these two putative SIMs, we mutated the two motifs individually or simultaneously for SUMO interaction. These SIM mutants and wild-type ORF45 plasmid were individually transfected into HEK293T cells and the cell lysates were tested in the GST pull-down assay for binding to GST or GST-SUMOs. As shown in Fig 2E, mutations of either SIMs abrogated the interaction between ORF45 and SUMO1, mutation of SIM1 impaired the binding of ORF45 to SUMO2, mutation of SIM2 completely blocked the interaction between ORF45 and SUMO2, and double mutations on both SIM1 and SIM2 completely abolished the interaction between ORF45 and SUMO1/2 (Fig 2E). These results indicated that KSHV ORF45 directly binds to SUMO1/2 via two SIMs.

### KSHV ORF45 acts as a SIM-dependent SUMO E3 ligase for RSK1

**Fig 3:**
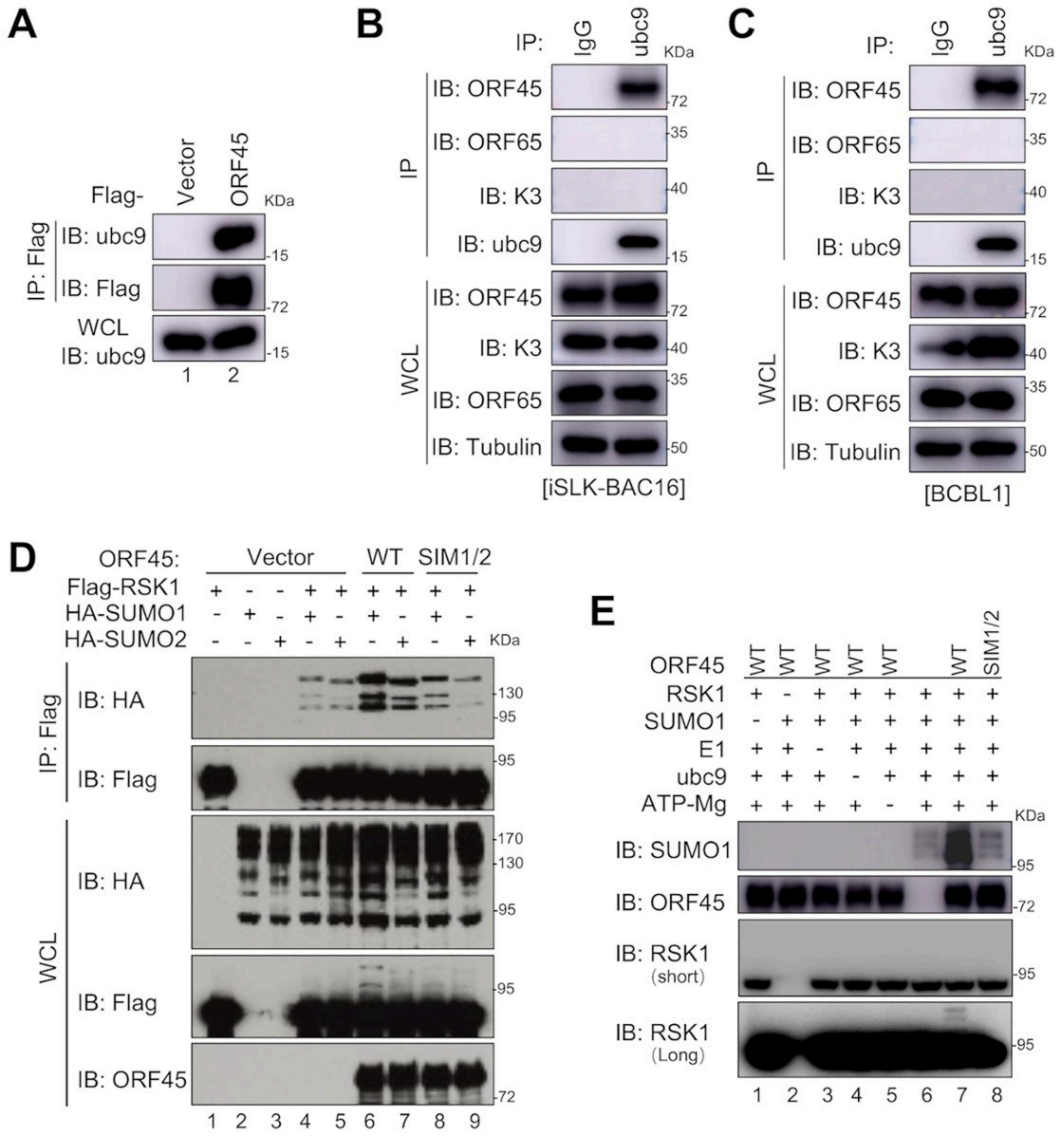
KSHV ORF45 acts as a SIM-dependent SUMO E3 ligase for RSK1. (**A**) KSHV ORF45 binds to endogenous ubc9. HEK293T cells were transfected with Flag-tagged ORF45 or vector control and cell lysates were subjected to IP and IB with indicated antibodies at 48 h post-transfection. (**B-C**) KSHV ORF45 binds to endogenous ubc9 during KSHV lytic replication. iSLK-BAC16 (B) and BCBL1 (C) cells were treated with doxycycline/sodium butyrate or TPA to induce KSHV lytic replication, respectively. Cell lysates were subjected to IP and IB with indicated antibodies at 72 h post-induction. (**D**) KSHV ORF45 enhances RSK1 SUMOylation via SIM1/2 in cells. HEK293T cells were transfected with indicated plasmids and cell lysates were subjected to denatured IP and IB with indicated antibodies at 48 h post-transfection. (**E**) KSHV ORF45 enhances RSK1 SUMOylation via SIM1/2 *in vitro*. Recombinant RSK1 was incubated with SAE1/UBA2 (E1), Ubc9 (E2), SUMO1, ATP/Mg, and ORF45 or ORF45^SIM1/2^ in SUMO reaction buffer. The SUMOylation of RSK1 was detected by indicated antibodies.

SUMO E3 ligases can interact with both SUMOs (SUMO1, SUMO2, or SUMO3) and E2 SUMO-conjugating enzyme ubc9, and facilitate the SUMOylation of the target proteins [14]. Since KSHV ORF45 binds to SUMO1/2 via SIMs and promotes RSK1 SUMOylation in cells, we next examined whether ORF45 may bind to ubc9 and function as a SUMO E3 ligase. Indeed, ORF45 bound to endogenous ubc9 in cells (Fig 3A), and importantly, ORF45-ubc9 associations were readily detectable during KSHV lytic replication in both iSLK-BAC16 and BCBL1 cells, while other lytic proteins, such as ORF65 and K3, cannot bind to ubc9 (Fig 3B and 3C). Furthermore, mutations on SIM1/2 abolished ORF45-mediated RSK1 SUMOylation in cells (Fig 3D, compare lane 6 with lane 8, and lane 7 with lane 9). Consistently, purified ORF45 enhanced RSK1 SUMOylation in the presence of SUMO1, E1, ubc9, and ATP/Mg *in vitro*, while mutations on SIM1/2 abrogated ORF45-mediated RSK1 SUMOylation (Fig 3E, compare lane 8 with lane 7), suggesting ORF45 is a SIM-dependent SUMO E3 ligase. These results demonstrated that KSHV ORF45 binds to SUMO1/2 via two SIMs and acts as a SUMO E3 ligase for RSK1 during KSHV lytic replication.

### KSHV ORF45 controls RSK1 phosphorylation and SUMOylation via two distinct functional regions

Our previous work identified that KSHV ORF45 interacts with and persistently activates cellular RSKs via amino acid (aa) 19–77 by forming high molecular mass protein complexes with RSK and ERK to prevent their dephosphorylation [12]. We also showed that ORF45 mutants with F66A or Δ19–77 failed to activate RSKs [5,6,12]. Here, we sought to determine whether RSK1 activation by ORF45 affects RSK1 SUMOylation and whether ORF45-mediated RSK1 SUMOylation affects RSK1 activation. To test this, we co-expressed ORF45^WT^, ORF45^Δ19–77^, ORF45^F66A^, or ORF45^SIM1/2^ with Flag-RSK1 in the presence of HA-SUMO1, and examined RSK1 SUMOylation and phosphorylation by specific antibodies. Consistent with previous finding [5,6], ORF45^WT^ stimulated both RSK1 phosphorylation at T359/S361 and RSK1 SUMOylation while ORF45^Δ19–77^ and ORF45^F66A^ were unable to activate RSK1 phosphorylation, and ORF45^SIM1/2^ failed to enhance RSK1 SUMOylation (S3 Fig). Interestingly, Δ19–77 or F66A mutations did not affect RSK1 SUMOylation by ORF45 and ORF45^SIM1/2^ mutant were still able to induce RSK1 phosphorylation to a comparable level as ORF45^WT^ (S3 Fig). These results indicated that KSHV ORF45 controls RSK1 phosphorylation and SUMOylation by two distinct functional motifs, F66A and SIMs, respectively, which do not affect each other.

### The SUMO E3 ligase activity of ORF45 is required for KSHV lytic replication

**Fig 4:**
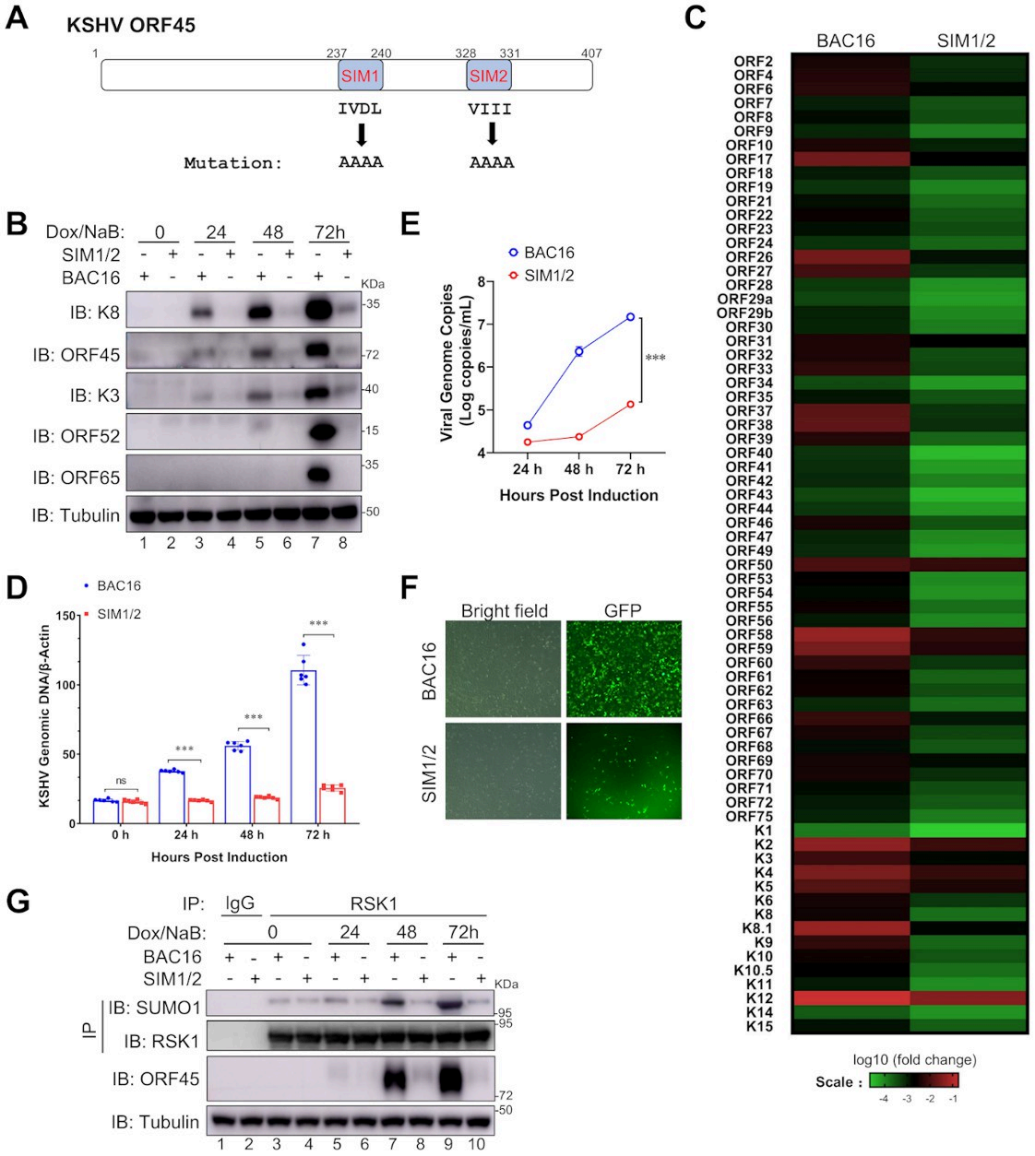
The SUMO E3 ligase activity of ORF45 is required for KSHV lytic replication. (**A**) Generation of BAC16^SIM1/2^ mutant (ORF45 SUMO E3 ligase defective mutant). (**B-C**) ORF45 SUMO E3 ligase activity is required for viral gene expressions at both protein and RNA levels during KSHV lytic replication. iSLK-BAC16 and iSLK-BAC16^SIM1/2^ cell lines were treated with doxycycline and sodium butyrate to induce KSHV lytic replication. Cell lysates were collected at indicated time points and subjected to IB with indicated antibodies (B). Total RNA was extracted, reverse-transcribed into cDNA, and used for KSHV whole-genome qPCR array analysis at 72 h post-induction. The Δ*C*_*T*_ values for each primer set were calculated and converted to a heatmap using R (C). (**D**) ORF45 SUMO E3 ligase activity is required for KSHV viral genomic DNA replication. iSLK-BAC16 and iSLK-BAC16^SIM1/2^ cell lines were induced with doxycycline and sodium butyrate and cell lysates were collected at indicated time point. Total DNA was isolated and viral genomic DNA was quantified by qPCR. (**E-F**) ORF45 SUMO E3 ligase activity is required for progeny virus production. iSLK-BAC16 and iSLK-BAC16^SIM1/2^ cell lines were induced with doxycycline and sodium butyrate and the culture medium containing progeny viruses were collected at indicated time point. Total DNA was isolated and viral genomic DNA was quantified by qPCR (E). HEK293A cells were infected by the supernatant containing progeny virus at 72 h post-induction and GFP level was determined by fluorescence microscope at 24 h post-infection (F). (**G**) RSK1 SUMOylation is impaired in SIM1/2 mutant virus during lytic replication. iSLK-BAC16 or iSLK-BAC16^SIM1/2^ cells were treated with doxycycline and sodium butyrate to induce lytic replication. The cell lysates were collected at indicated time points and subjected to denatured IP and IB with indicated antibodies. Mean ± SD; n = 5; ***p<0.001 by Student’s *t*-test in (D) and (E).

To further investigate the role of ORF45 SIMs for KSHV lytic replication, we introduced mutations of both SIM1 and SIM2 into KSHV BACmid (BAC16) (Fig 4A and S4A and S4B Fig) and generated the iSLK-BAC16^SIM1/2^ cell line, in which RTA expression is controlled by a Tet-On promoter [15]. With the treatment of doxycycline and sodium butyrate, KSHV switches from latency to lytic replication in iSLK cells. We first examined the expression levels of viral proteins by western blot. In iSLK-BAC16 cells, the protein expression levels of immediate-early genes (K8 and ORF45), early genes (K3 and ORF52), and the late gene (ORF65) were detectable at 24 h post-induction and continuously increased at 48 h and 72 h post-induction. However, the expressions of these proteins dramatically decreased in iSLK-BAC16^SIM1/2^ cells (Fig 4B). To systematically compare the KSHV viral gene expression profile between iSLK-BAC16 and iSLK-BAC16^SIM1/2^ cells, we next examined viral mRNA levels by a genome-wide quantitative RT-PCR array at 72 h post lytic replication. As shown in Fig 4C, mutation on SIMs of ORF45 dramatically reduced the overall mRNA levels of KSHV genes in iSLK-BAC16^SIM1/2^ cells as compared to iSLK-BAC16 cells, suggesting that the SUMO E3 ligase activity of ORF45 is critical for KSHV lytic replication (Fig 4C). Consistently, viral DNA copy number also significantly decreased in iSLK-BAC16^SIM1/2^ cells (Fig 4D). Last, we evaluated the progeny virus production by quantifying the viral genomic DNA levels in the culture supernatant or quantifying the GFP level of progeny virus-infected cells and found that SIM1/2 mutation on ORF45 dampened the production of infectious progeny virus during KSHV lytic replication (Fig 4E and 4F and S4C Fig). Importantly, SIM1/2 revertant mutation totally rescued these replication defects (S5A–S5I Fig), suggesting ORF45 SIM1/2 play critical role for KSHV lytic replication. In addition, RSK1 SUMOylation level did not increase in iSLK-BAC16^SIM1/2^ cell as compared to iSLK-BAC16 cells upon KSHV lytic replication (Fig 4G). These results suggested that the SIM-dependent SUMO E3 ligase activity of ORF45 plays a critical role in KSHV lytic replication and progeny virus production.

### The SUMO E3 ligase activity is conserved in RRV ORF45

**Fig 5:**
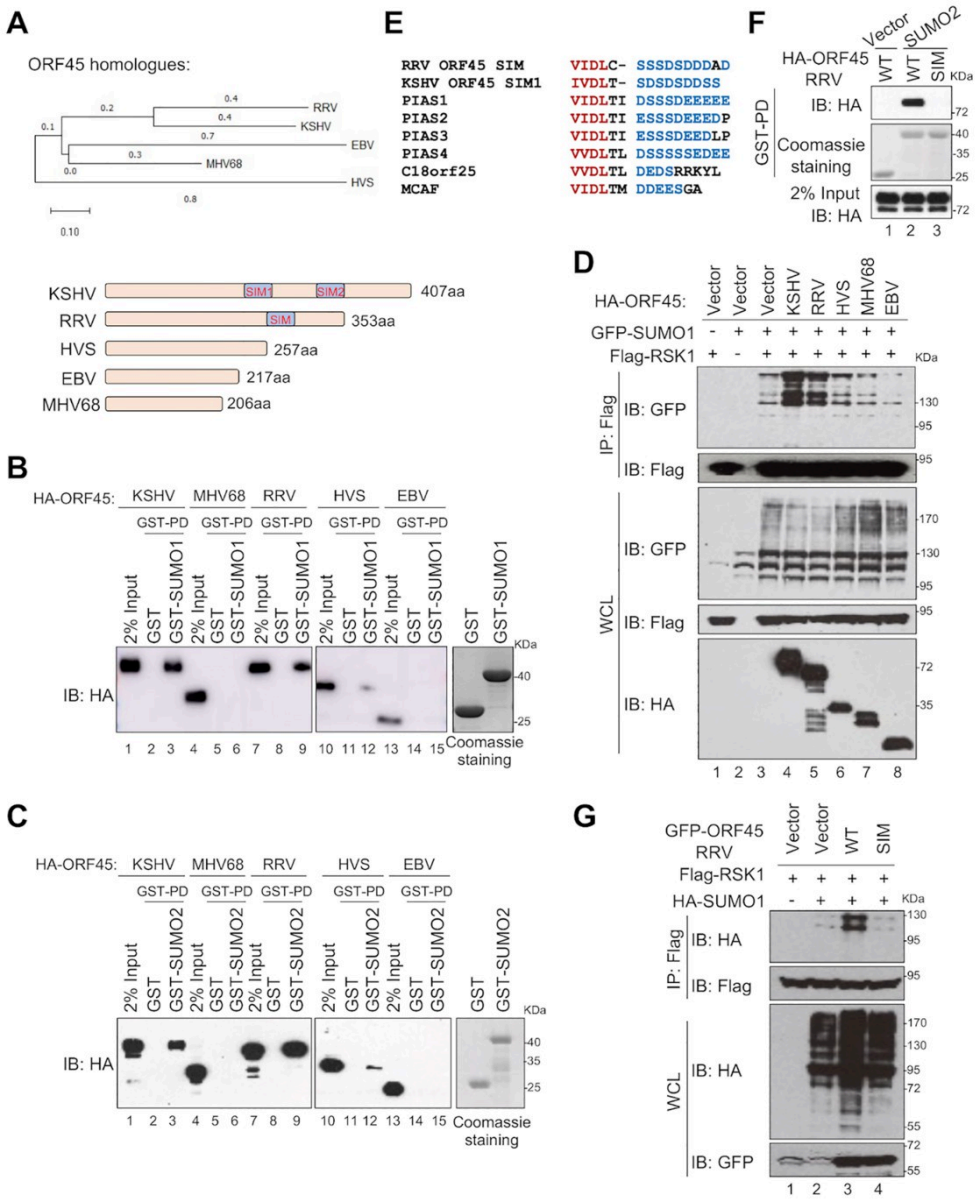
The SUMO E3 ligase activity is conserved in ORF45 of RRV. (**A**) Phylogenetic tree of the indicated γ-herpesviruses based on their ORF45 sequence. Scale: 0.1 (upper panel). Schematic diagram for ORF45 homologues (lower panel). (**B-C**) The interaction between ORF45 homologues and SUMO1 (B) or SUMO2 (C). GST only, GST-SUMO1, or GST-SUMO2 were mixed with the cell lysates from HEK293T transfected with ORF45^KSHV^, ORF45^RRV^, ORF45^HVS^, ORF45^EBV^, or ORF45^MHV68^ for 30 min and the mixture was subjected to GST pull-down assay. (**D**) The effects of ORF45 homologues on RSK1 SUMOylation. HEK293T cells were transfected with indicated plasmids and cell lysates were subjected to denatured IP and IB with indicated antibodies at 48 h post-transfection. (**E**) RRV ORF45 only contains one SIM. Alignment between ORF45^RRV^ SIM and well-characterized SIMs from indicated genes. Red: core sequence; Blue: acidic amino acid tail. (**F**) SIM mutation abolished RRV ORF45-SUMO2 interaction. (**G**) SIM mutation abrogated RRV ORF45-mediated RSK1 SUMOylation.

Finally, we determined whether SUMO E3 ligase activity is a common signature of ORF45 homologs in other γ-herpesviruses. ORF45 is less conserved among γ-herpesviruses and varies in protein length. Among these ORF45 homologs, ORF45^KSHV^ is the longest one with 407 aa. ORF45^RRV^, ORF45^HVS^, and ORF45^EBV^ are 353 aa, 257 aa, and 217 aa, respectively, while ORF45^MHV68^ is the shortest one with only 206 aa (Fig 5A). We first compared SUMO binding among these ORF45 homologs. As shown in Fig 5B and 5C, only ORF45^KSHV^ and ORF45^RRV^ were strongly bound to SUMO1 or SUMO2, while HVS^ORF45^ was slightly bound to SUMO1 or SUMO2, and the ORF45^MHV68^ and ORF45^EBV^ showed no interaction with SUMO1 or SUMO2 (Fig 5B and 5C). Next, we compared these ORF45 homologs for RSK1 SUMOylation. We found that only ORF45^KSHV^ and ORF45^RRV^ could potentiate RSK1 SUMOylation, while ORF45^HVS^, ORF45^MHV68^, and ORF45^EBV^ had little or no effects on RSK1 SUMOylation (Fig 5D). Sequence analysis showed that ORF45^RRV^ contains one SIM (_259_VIDL_262_) (Fig 5E), and SIM mutation abolished ORF45^RRV^-SUMO2 interaction (Fig 5F). Importantly, SIM mutation also abrogated ORF45^RRV^-mediated RSK1 SUMOylation (Fig 5G). These results demonstrated that the SUMO E3 ligase activity is not conserved in the ORF45 homologues of γ-herpesviruses and only ORF45^RRV^ also works as a SUMO E3 ligase for RSK1 SUMOylation with a similar mechanism. Taken together, we characterized that KSHV ORF45 acts as a novel SIM-dependent SUMO E3 ligase for RSK1, which is conserved in RRV. The SUMO E3 ligase activity of ORF45 is required for KSHV lytic replication.

## Discussion

SUMOylation delicately regulates various cellular signaling pathways, which depend on the substrate specificity of SUMO E3 ligase. To successfully establish the persistent infection, viruses encode their own E3 ligases to manipulate host SUMOylation and ubiquitination processes. The adenovirus early region 4-ORF3 protein works as a SUMO E3 ligase to promote TIF-1γ SUMOylation and proteasomal degradation [16]; SM protein of EBV, UL54/ICP27 of herpes simplex virus 1 (HSV-1), and UL69 of cytomegalovirus bind to ubc9 and have SUMO E3 ligase activity to globally upregulate cellular SUMOylated proteins [17]; ICP0 of HSV-1 is identified as a ubiquitin E3 ligase to disrupt PML nuclear body [18], and NSs of Toscana virus contains ubiquitin E3 ligase activity to facilitate RIG-I ubiquitination and proteasome-dependent degradation [19]. KSHV also employs several viral proteins, such as LANA, RTA, and K8, to modulate the host SUMO system [20–23]. Here, we identified that KSHV ORF45 acts as a SIM-dependent SUMO E3 ligase to modify RSK1 during KSHV lytic replication. Post-translational modifications delicately regulate RSK1 functions: phosphorylation on multiple serine/threonine residues (T359, S363, S380, and T573) regulates RSK1 kinase activity [24], while SUMOylation on K110, K335, and K421 determines RSK1 substrate specificity via SUMO-SIM association [7]. RSK1 phosphorylates eIF4B to promote protein translation, which is required for efficient lytic replication of KSHV [7]. eIF4B has one SIM (_166_IRVDV_170_) that binds to SUMOylated RSK1 via SUMO-SIM association and mutation of SIM drastically reduces RSK1-eIF4B interaction [7]. To activate the RSK1-eIF4B axis, KSHV encodes ORF45 to manipulate both phosphorylation and SUMOylation of RSK1 via two distinct motifs: ORF45 binds to RSK1 via aa 17–90 region and promotes RSK1 phosphorylation [6], while ORF45 binds to SUMOs via two SIMs to facilitate RSK1 SUMOylation. Interestingly, aa 17–90 and SIMs of ORF45 do not affect each other, and mutation on either one will dramatically dampen KSHV lytic replication, suggesting the essential role of the ORF45-RSK-eIF4B axis for KSHV lytic replication.

Due to the relatively small genome, viruses have to condense more genetic information into their limited genome. KSHV ORF45 has 407 amino acids but does not contain any classic domain. However, it utilizes multiple small motifs to target various cellular or viral proteins, benefiting the KSHV life cycle. ORF45 binds to ORF33 and USP7 via aa 383–407 and _223_EGPS_226_ (USP7-binding motif: P/A/E-G-X-S), respectively, and stabilizes ORF33 via USP7-mediated deubiquitination [25]; ORF45 binds to RSK1/2 via aa 17–90 and activates RSK1/2 by preventing them from dephosphorylation [5,12]; ORF45 binds to SUMOs via two SIMs to regulate RSK1 SUMOylation; ORF45 is mono-ubiquitinated at K297 that mediates the association with lipid rafts and promotes viral assembly and egress [9]; and ORF45 utilizes its S41 and S162 to compete with IRF7 for TBK1/IKKε-mediated phosphorylation, leading blockage of type I IFN production [10]. Unlike ORF45^KSHV^, the ORF45 homologs from other γ-herpesviruses are shorter in length (353 aa for ORF45^RRV^, 257 aa for ORF45^HVS^, and 217 aa for ORF45^EBV^, 206 aa for ORF45^MHV68^) and the capability of SUMO interaction is not conserved among these ORF45 homologs (Fig 5A). ORF45^KSHV^ has two SIMs, ORF45^RRV^ has only one SIM, while ORF45^HVS^, ORF45^MHV68^, and ORF45^EBV^ have no SIM. Consistently, only ORF45^KSHV^ and ORF45^RRV^ can promote SUMOylation of RSK1, while others cannot, indicating that SIM-dependent SUMO E3 ligase activity emerges on ORF45 during herpesvirus evolution and is essential for KSHV lytic replication. RSK1 is a bona fide substrate for the SUMO E3 ligase activity of KSHV ORF45 and further studies are required to explore the cellular and viral substrate diversity of ORF45 during KSHV lytic replication.

## Methods

### Antibodies and chemicals

Anti-RSK1 (#9333S) antibody was ordered from Cell Signaling Technology. HRP anti-HA (#901519), HRP anti-Flag (#637311) antibodies were purchased from BioLegend. pRSK1 (Thr359/Ser363) (#AP0539), SUMO1 (#A19121), SUMO2/3 (#A2571), and ubc9 (#A4396) were purchased from ABclonal. Mouse anti-GFP (#sc-9996) antibody was ordered from Santa Cruz Biotechnology. Anti-RTA (ORF50) monoclonal mouse antibody was given by Dr. Ke Lan (Wuhan University, China) [26]. Anti-ORF65 monoclonal mouse antibody was given by Dr. Shou-Jiang Gao (University of Pittsburgh, USA) [27]. Monoclonal antibodies against ORF45 and ORF52 and polyclonal antibodies against K3 and K8 were described previously [10,28,29]. EZview Red Anti-Flag M2 Affinity Gel (#F2426), TPA (#P1585) and sodium butyrate (#B5887) were ordered from Sigma, Doxycycline (#S4163) was ordered from Selleck, Glutathione Sepharose 4B (#17-0756-01) was ordered from GE Healthcare. ClonExpress II One Step Cloning Kit (#C122-01) and HiScript II Q RT SuperMix for qPCR (+gDNA wiper) (#R223-01) were purchased from Vazyme Biotech. FuGENE HD Transfection Reagent (#E2311) was ordered from Promega. Lipofectamine 3000 (#3000015) were purchased from Thermo Fisher Scientific.

### Cells

HEK293T and HEK293A cells were cultured in Dulbecco’s modified Eagle’s medium (DMEM) containing 10% FBS and antibiotics. BJAB cells were cultured in RPMI 1640 containing 10% FBS and antibiotics. BCBL1 cells were given by Dr. Ersheng Kuang (Sun Yat-Sen University, China) and were cultured in RPMI 1640 containing 20% FBS and antibiotics. iSLK-BAC16, iSLK-BAC16^ΔORF45^, and iSLK-BAC16^SIM1/2^ cells were cultured in DMEM containing 10% FBS, antibiotics, puromycin (1 μg/ml), G418 (250 μg/ml), and hygromycin (400 μg/ml). Doxycycline (2 μg/ml) and sodium butyrate (1 mM) treatment was used to induce KSHV lytic replication in iSLK-BAC16 cells.

### Plasmid constructs

KSHV genes were cloned into pLVX or pCDH vector using BAC16 as a template. The cDNA for human RSK1, RSK2, SUMO1, and SUMO2 were obtained from the Core Facility of Basic Medical Sciences, Shanghai Jiao Tong University School of Medicine. pCR3.1-ORF45 is originally cloned by Dr. Fanxiu Zhu (Florida State University, USA). ORF45 homologues from RRV, HVS, MHV68 or EBV were synthesized by GenScript. The mutations of ORF45, SUMO1, or SUMO2 were generated using ClonExpress II One Step Cloning Kit (Vazyme Biotech, #C122-01). RSK1, RSK2, ORF45^KSHV^, ORF45^RRV^, ORF45^HVS^, ORF45^MHV68^, ORF45^EBV^, ORF45^F66A^, ORF45^SIM1/2^, SUMO1, SUMO1^G/A^, SUMO2, and SUMO2^G/A^ were subcloned into pKH3, pCMV-3Flag, pEGFP-C1, pLVX-3Flag, or pCDH-3Flag vectors as indicated. All constructs were sequenced using an ABI PRISM 377 automatic DNA sequencer to verify 100% correspondence with the original sequence.

### Yeast two-hybrid screening

The yeast two-hybrid screen was performed as described previously [11]. The KSHV ORF45 bait plasmid was constructed by cloning the full-length coding sequence of ORF45 in pAS2–1 in frame with GAL4 DNA-binding domain (DBD). The yeast strain Y190 carrying the plasmid pAS2–1-ORF45 was used to screen a human lymphocyte Matchmaker cDNA library (Clontech). One million transformants were plated on Leu^−^Trp^−^His^−^ plus 50 mM 3-amino-1,2,4-triazole (3-AT) plates. His^+^LacZ^+^ colonies were picked up for sequencing and further analyses.

### Generation of ORF45SIM1/2 mutant on KSHV BAC16 genome

The mutagenesis of BAC16 [30] was performed using a recombineering system as described by Tischer et al. [31,32]. Briefly, the Kan/I-SceI cassettes were amplified from pEPKan-S plasmid by PCR with primers ORF45-SIM1-5’ (5’-agggccctcgtggcgccccctgcgcgccgcacccaccgcggccgccgacgcgacatcggactctgatagcgaggatgacgacgataagtaggg-3’) and ORF45-SIM1-3’ (5’- cggagagttggaactgtcatcgctatcagagtccgatgtcgcgtcggcggccgcggtgggtgcggcgcgcacaaccaattaaccaattctgattag-3’) for SIM1 mutation and primers ORF45-SIM2-5’ (5’-cagagacaaccaaacttaagccgcaaagcagtggcgtccgcggcagctgcatcctcggggagtgacacagaggatgacgacgataagtaggg-3’) and ORF45-SIM2-3’ (5’- ggcggacgagggctcctcgtctgtgtcactccccgaggatgcagctgccgcggacgccactgctttgcggcaaccaattaaccaattctgattag-3’) for SIM2 mutation. The purified PCR fragments were electroporated into BAC16-containing GS1783 cells that had been induced at 42°C for 15 min. The recombinant clones were selected at 32°C on LB plates containing 34 μg/ml chloramphenicol and 50 μg/ml kanamycin and then analyzed by restriction enzyme digestion. Positive clones were cultured with 1% L-arabinose, induced at 42°C again, and plated on LB plates containing 1% L-arabinose for secondary recombination. Colonies which survived on the L-arabinose plates were replicated on plates with 34 μg/ml chloramphenicol alone and on plates with both 34 μg/ml chloramphenicol and 50 μg/ml kanamycin. The kanamycin-sensitive clones were analyzed by restriction enzyme digestion, and proper mutations were further confirmed by DNA sequencing.

### Generation of iSLK cell line containing modified KSHV genome

iSLK cells seeded in a 24-well plate were transfected with 1 μg BAC DNA by FuGENE HD (Promega). Cells were subcultured into a T150 flask and selected with 1 μg/ml puromycin, 500 μg/ml hygromycin, and 450 μg/ml G418 at 48 h post-transfection. The resistant colonies containing modified KSHV genome will be generated around 12 days post-selection.

### *In vitro* SUMOylation assay

*In vitro* SUMOylation assay was performed as described previously [7,33]. SUMO1 (#K-700), SAE1/UBA2 (#E1-315), ubc9 recombinant proteins (#E2-645), and SUMO conjugation reaction buffer kit (#SK-15) were purchased from Boston Biochem. Flag-RSK1, Flag-ORF45, or Flag-ORF45^SIM^ were purified from transfected HEK293T cells by affinity purification via anti-Flag M2 affinity Gel (Sigma, #F2426). 1 μg purified RSK1 substrate was mixed with 2 μg SUMO1, 200 ng SAE1/UBA2, 100 ng ubc9, 1 μg ORF45 or ORF45^SIM1/2^, 1 x ATP/Mg, and 1 x SUMO reaction buffer in 20 μl reaction system for 3 h at 30°C and stopped by adding stop buffer. The reaction mixture was subjected to standard immunoblotting analyses to detect the SUMOylation of RSK1.

### Immunoprecipitation and Immunoblotting

For co-immunoprecipitation, the transfected HEK293T cells, doxycycline/sodium butyrate-induced iSLK-BAC16 cells, or TPA-induced BCBL1 cells were collected and washed twice with cold phosphate-buffered saline (PBS) and lysed in a whole cell lysis buffer (WCL) containing (50 mM Tris·HCl [pH 7.4], 150 mM NaCl, 1% NP-40, 1 mM EDTA, 10% glycerol, protease inhibitor cocktail [Roche]) for 20 min on ice. The cell lysates were then centrifuged at 15,000 rpm for 15 min and the clear supernatants were subjected to immunoprecipitation with indicated specific antibodies or control IgG with protein A/G agarose (ThermoFisher Scientific, #20422) by the standard protocol. After overnight incubation at 4°C, the beads were washed for three times with WCL and twice with PBS, and then boiled with the 2 x SDS loading buffer for 10 min. The immunoprecipitants were applied to standard immunoblotting analyses with specific antibodies.

Immunoprecipitation in denaturing conditions for detecting SUMOylated protein was described previously [33]. Briefly, cells were lysed in SDS lysis buffer made by 1:3 ratio of Buffer I (5% SDS, 0.15 M Tris-HCl pH 6.8, 30% glycerol) and Buffer II (25 mM Tris-HCl pH 8.3, 50 mM NaCl, 0.5% NP-40, 0.5% deoxycolate, 0.1% SDS, 1 mM EDTA) supplemented with protease inhibitors cocktail (Roche), 1 mM DTT and 5 mM N-Ethylmaleimide (NEM, Sigma) and denatured 5 min at 95°C. Cell lysates were then centrifuged at maximum speed for 10 min. Supernatants were either directly resolved by SDS-PAGE or diluted 1:5 in E1A buffer (50 mM Hepes pH 7.5, 250 mM NaCl, 0.1% NP-40, 1 mM EDTA, supplemented with protease inhibitors cocktail, 1 mM DTT and 5 mM NEM) and then immunoprecipitated using anti-Flag M2 affinity Gel (Sigma, #F2426). After 4 h incubation at 4°C, the beads were washed for three times with WCL and twice with PBS, and then boiled with the 2 x SDS loading buffer for 10 min. The immunoprecipitants were applied to standard immunoblotting analyses with specific antibodies.

### RNA purification and RT-qPCR

Total RNA was extracted from cells with TRIzol reagent (Sigma) according to the manufacturer’s protocol. Afterwards 0.5 μg of total RNA was reverse transcribed by HiScript II Q RT SuperMix for qPCR (+gDNA wiper) (Vazyme Biotech, #R223-01) and the cDNA was quantified by SYBR green based qPCR using gene specific primers. The relative level of gene expression was calculated by the 2^-ΔCt^ and the ΔΔCt methods, where GAPDH was used for normalization. The RT-qPCR graphs represent the average of at least three independent experiments. The sequences of the primers used in RT-qPCR have been described previously [7].

### Measurement of viral DNA copy number during KSHV lytic replication

iSLK-BAC16 or iSLK-BAC16^SIM1/2^ cells were uninduced or induced with doxycycline (2 μg/ml) and sodium butyrate (1 mM), and lysed in RIPA buffer followed by sonication and then centrifugation to remove cell debris. Total DNA was purified from the supernatant by phenol-chloroform extraction and 10 ng of total DNA was analyzed in qPCR. The viral DNA was measured by qPCR using primers for ORF11 (Fw-GGCACCATACAGCTTCTACGA and Rev-CGTTTACTACTGCACACTGCA). The amount of viral DNA was normalized for the cellular DNA input, which was measured by qPCR specific for the β-actin genomic region (Fw-CGGGAAATCGTGCGTGACATT; Rev-CAGGAAGGAAGGCTGGAAGAGTG).

### Quantification of extracellular virion genomic DNA by real-time qPCR

iSLK-BAC16 or iSLK-BAC16^SIM1/2^ cells were uninduced or induced with doxycycline (2 μg/ml) and sodium butyrate (1 mM), and viral DNA was isolated from supernatant medium as previously described [7,34]. Briefly, medium from the infected cells was centrifuged to remove any cellular debris and treated with TurboDNase (Ambion) to remove any unprotected DNA. The viral particles were lysed with buffer AL (Qiagen), and the proteins were degraded with protease K (Qiagen). The DNA was then extracted using phenol-chloroform extraction and analyzed by SYBR green real-time PCRs with KSHV-specific primers ORF11 described above. Viral DNA copy numbers were calculated with external standards of known concentrations of serially diluted BAC16 DNA ranging from 1 to 10^7^ genome copies per reaction.

### GST pull-down assay

HEK293T cells were transfected with ORF45 homologues. At 48 h post-transfection, cells were harvested and lysed in a buffer containing 20 mM Tris-HCl at pH 7.5, 0.5% NP-40, 150 mM NaCl and protease inhibitors. The GST-tagged wild-type or mutant SUMO1 or SUMO2 or GST control proteins were purified by Glutathione Sepharose 4B (GE Healthcare, #17-0756-01), followed by incubation of WCL from cells transiently expressing ORF45 for 2 h at 4 °C. After washing three times with lysis buffer and twice with PBS, the beads were heated in 2 × SDS loading sample buffer at 95°C for 10 min and the coprecipitated proteins were analyzed by immunoblotting with indicated antibodies.

### Quantification and statistical analysis

All data were expressed as mean ± SD, unless otherwise noted. For parametric analysis, the *F* test was used to determine the equality of variances between the groups compared; statistical significance across two groups was tested by Student’s *t*-test; one-way analysis of variance (ANOVA) followed by Bonferroni’s *post hoc* test were used to determine statistically significant differences between multiple groups. *P*-values of less than 0.05 were considered significant.

## Supporting information

S1 Fig Screening of KSHV-encoded proteins for RSK1 SUMOylation. KSHV ORF45 enhances RSK1 SUMOylation. Individual KSHV-encoded gene or vector control was co-expressed with HA-RSK1 and denatured IP and IB were performed at 48 h post-transfection.
(TIF)

S2 Fig KSHV ORF45 specifically enhances RSK1 SUMOylation. (**A**) ORF45 promotes the SUMOylation of endogenous RSK1. BJAB cells stably expressing doxycycline-induced ORF45 were treated with or without doxycycline for 48 h and cell lysates were subjected to denatured IP and IB with indicated antibodies. (**B**) KSHV K8 cannot promote RSK1 SUMOylation. HEK293T cells were transfected with indicated plasmids and cell lysates were subjected to denatured IP and IB with indicated antibodies at 48 h post-transfection.
(TIF)

S3 Fig KSHV ORF45 controls RSK1 phosphorylation and SUMOylation via two separated functional motifs. HEK293T cells were transfected with indicated plasmids and cell lysates were subjected to denatured IP and IB with indicated antibodies at 48 h post-transfection.
(TIF)

S4 Fig Generation of ORF45 SUMO E3 ligase defective KSHV BAC. (**A-B**) Generation of BAC16^SIM1/2^ mutant by two-step recombination. The sequence of mutated SIM1 and SIM2 regions (A). SalI digestion patterns of wild-type BAC16 and BAC16^SIM1/2^ were determined by agarose gel electrophoresis (B). (**C**) KSHV ORF45 SUMO E3 ligase activity is required for progeny virus production. iSLK-BAC16 and iSLK-BAC16^SIM1/2^ cell lines were induced with doxycycline and sodium butyrate. The culture medium containing progeny viruses were collected at 72 h post-induction and used to infect HEK293A cells. The progeny virus infectivity was determined by quantification of GFP level by flow cytometry.
(TIF)

S5 Fig ORF45 SIM1/2 play critical roles for KSHV lytic replication. (**A-B**) Generation of SIM1/2^Rev^ from BAC16^SIM1/2^ by two-step recombination. The mutated regions were confirmed by DNA sequencing (A) and SalI digestion patterns were determined by agarose gel electrophoresis (B). (**C-I**) iSLK-BAC16, iSLK-SIM1/2, or iSLK-SIM1/2^Rev^ cells were treated with doxycycline and sodium butyrate to induce lytic replication. At 72 h post-induction, total RNA was extracted and used to evaluate the lytic gene expression by indicated primers (C-H). Total DNA was isolated from culture medium at indicated time point and viral genomic DNA was quantified by qPCR (I).
(TIF)
