# Supplementary figures and images for: The SUMO E3 ligase activity of ORF45 determines KSHV lytic replication
Source: PLoS Pathog. 2022 Apr 28;18(4):e1010504. doi: 10.1371/journal.ppat.1010504 (PMC9089915; doi:10.1371/journal.ppat.1010504)

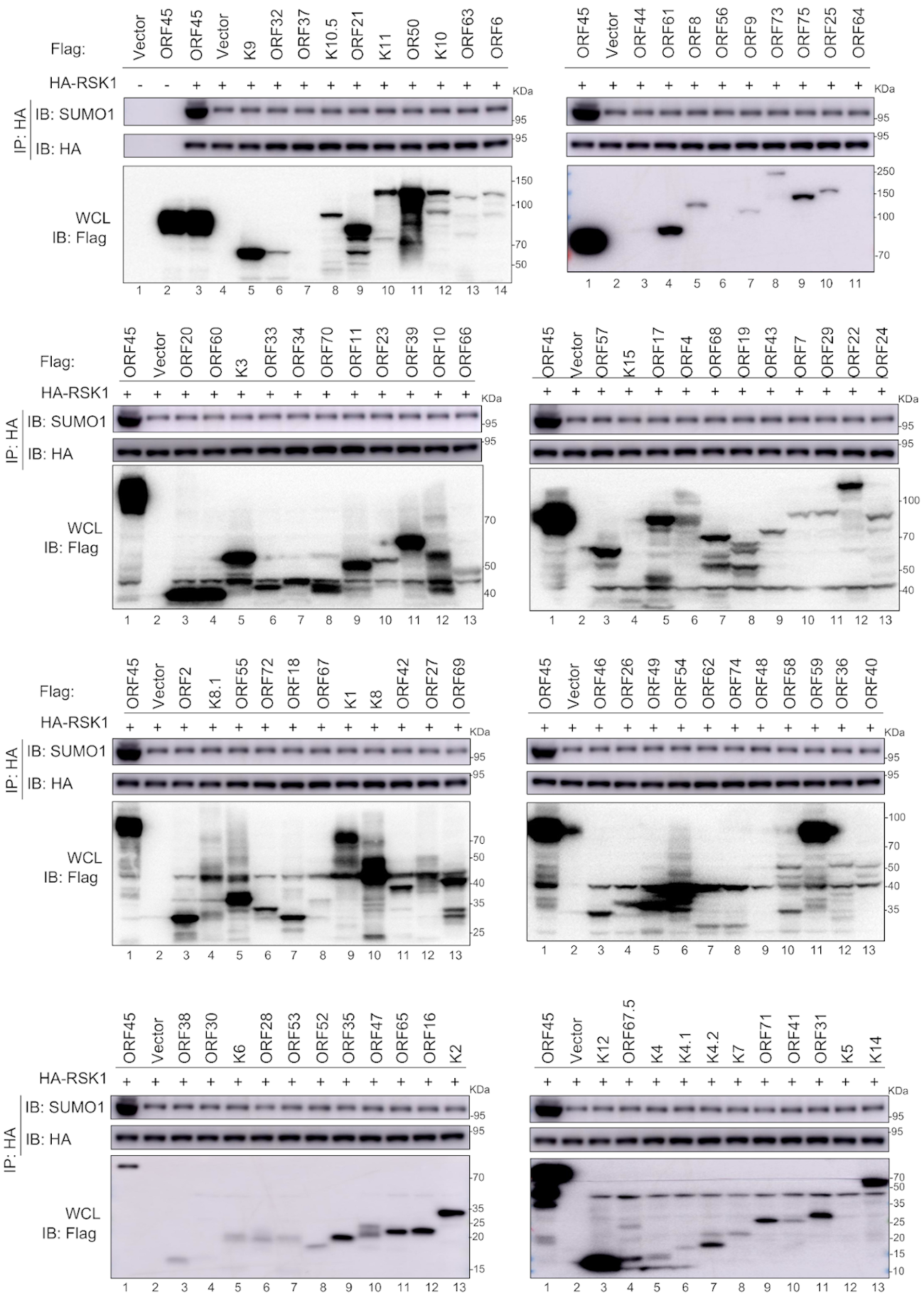

Supplement: S1 Fig — KSHV ORF45 enhances RSK1 SUMOylation. Individual KSHV-encoded gene or vector control was co-expressed with HA-RSK1 and denatured IP and IB were performed at 48 h post-transfection. (TIF) [file ppat.1010504.s001.tif]

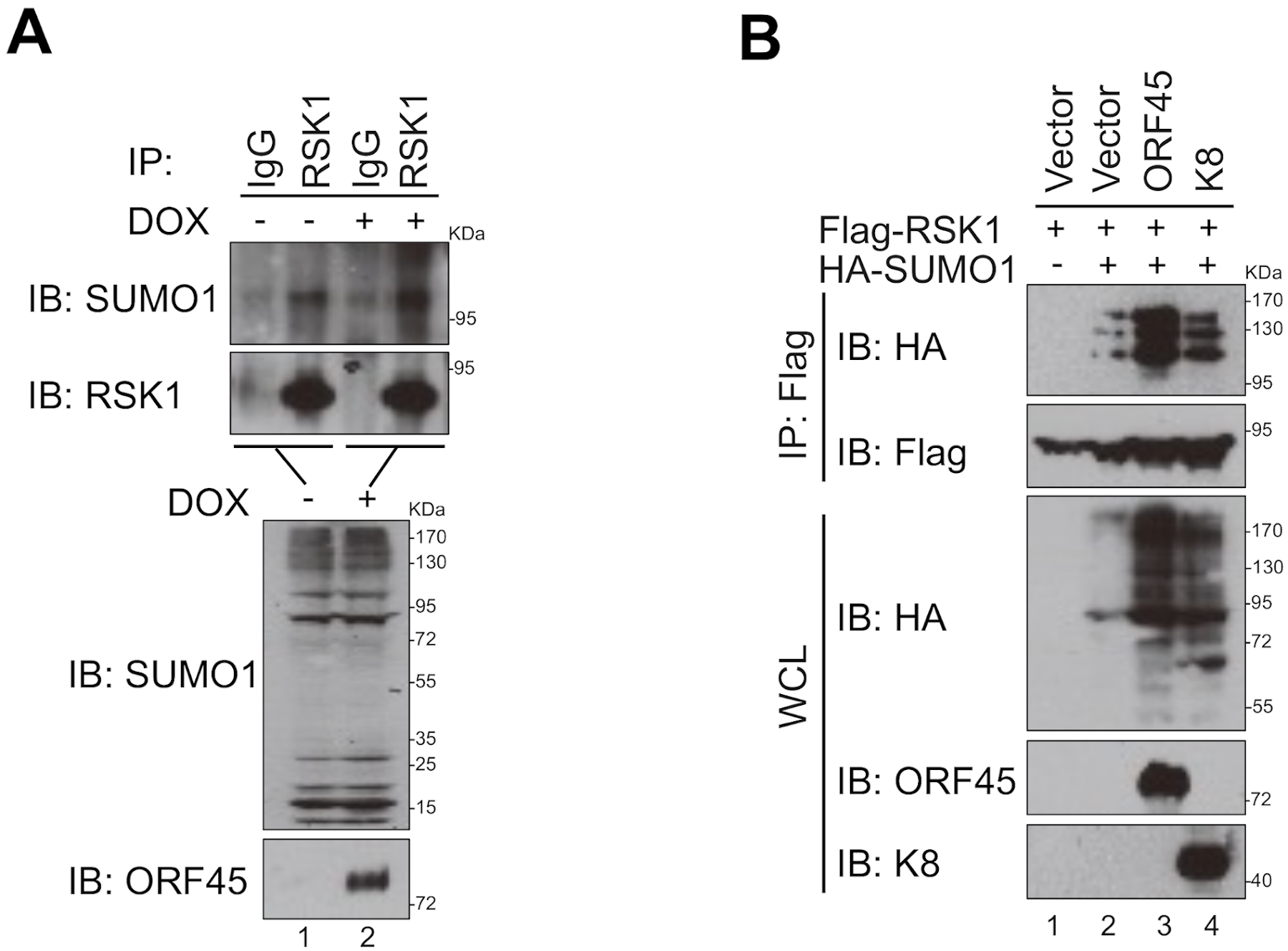

Supplement: S2 Fig — (A) ORF45 promotes the SUMOylation of endogenous RSK1. BJAB cells stably expressing doxycycline-induced ORF45 were treated with or without doxycycline for 48 h and cell lysates were subjected to denatured IP and IB with indicated antibodies. (B) KSHV K8 cannot promote RSK1 SUMOylation. HEK293T cells were transfected with indicated plasmids and cell lysates were subjected to denatured IP and IB with indicated antibodies at 48 h post-transfection. (TIF) [file ppat.1010504.s002.tif]

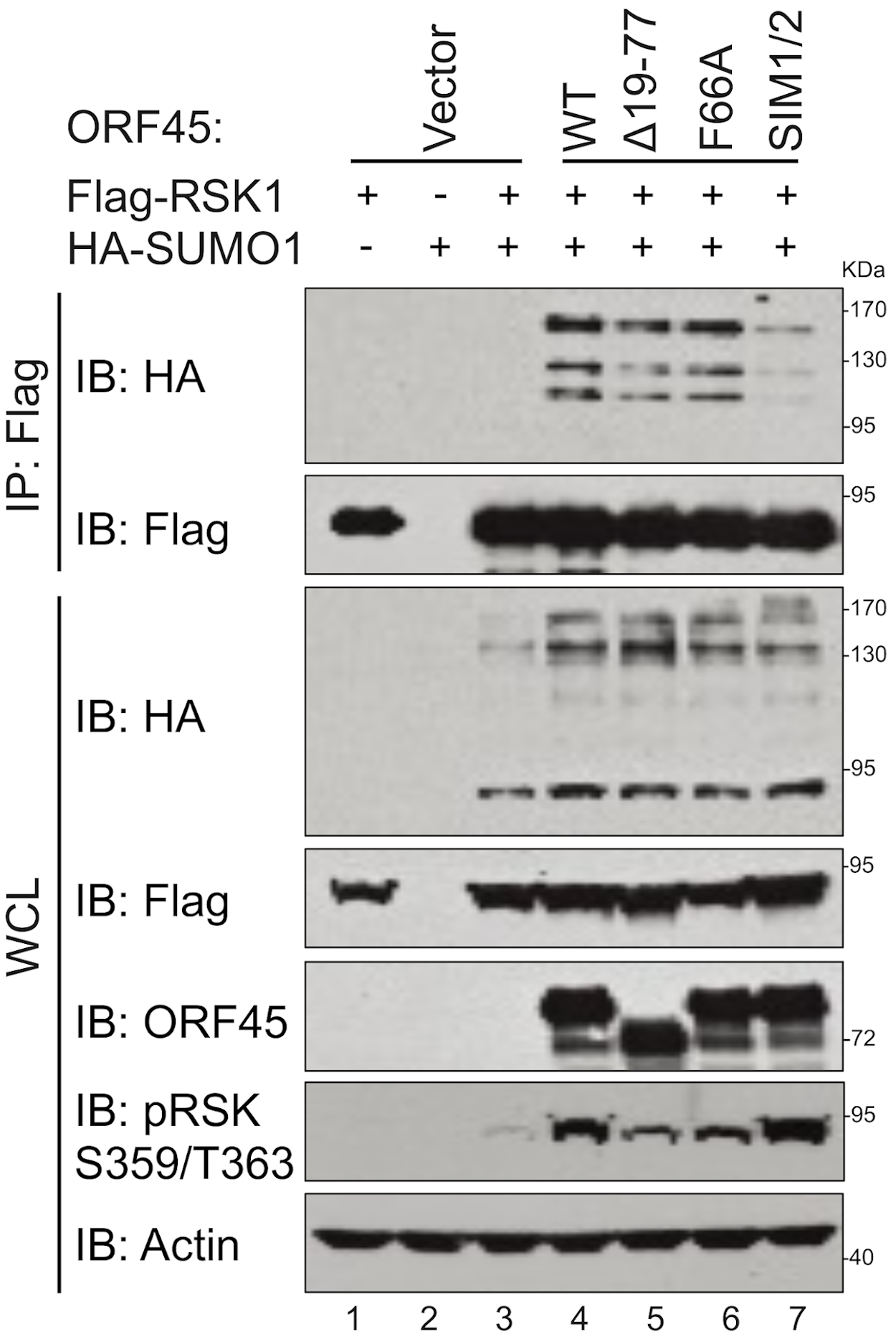

Supplement: S3 Fig — HEK293T cells were transfected with indicated plasmids and cell lysates were subjected to denatured IP and IB with indicated antibodies at 48 h post-transfection. (TIF) [file ppat.1010504.s003.tif]

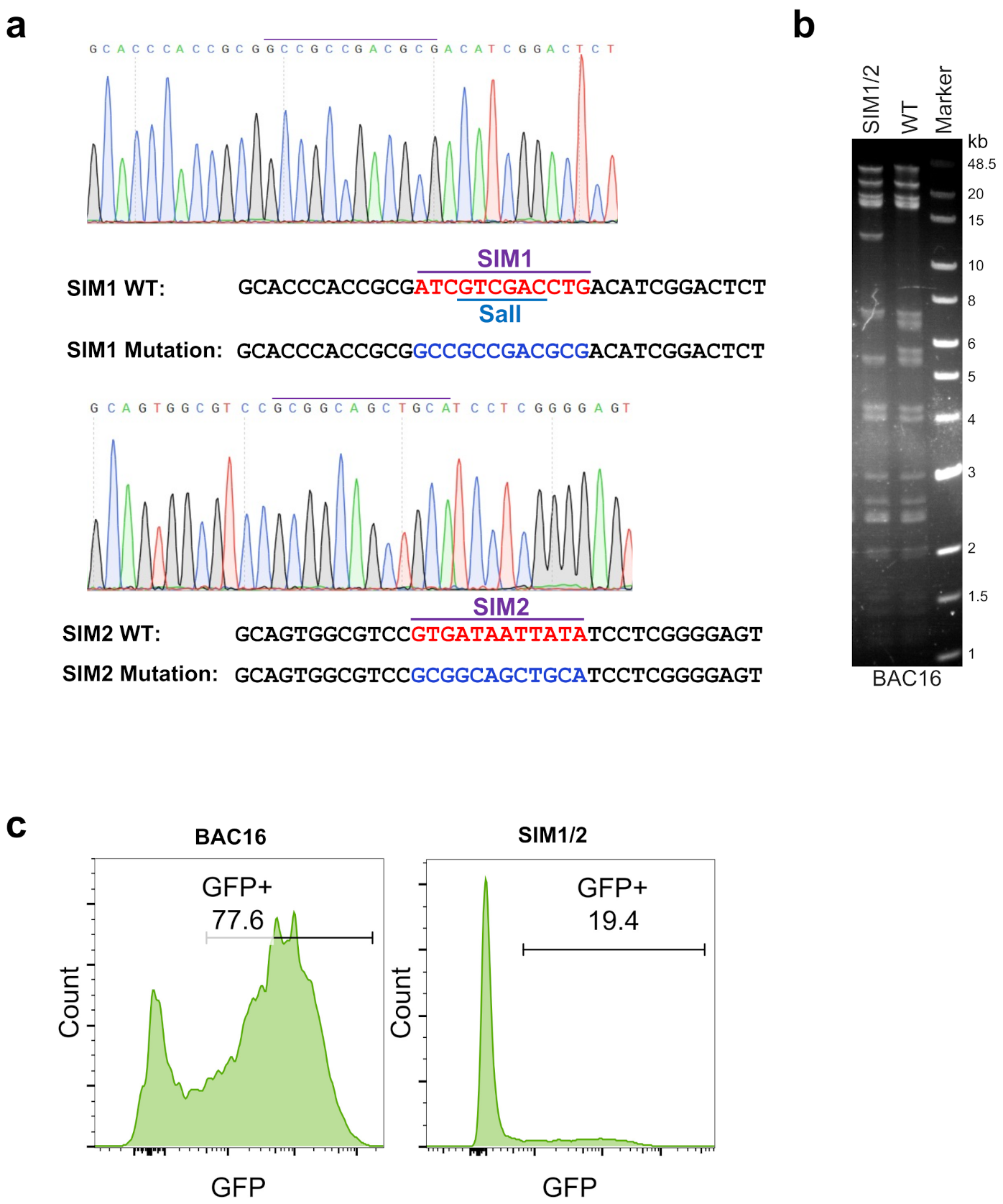

Supplement: S4 Fig — (A-B) Generation of BAC16SIM1/2 mutant by two-step recombination. The sequence of mutated SIM1 and SIM2 regions (A). SalI digestion patterns of wild-type BAC16 and BAC16SIM1/2 were determined by agarose gel electrophoresis (B). (C) KSHV ORF45 SUMO E3 ligase activity is required for progeny virus production. iSLK-BAC16 and iSLK-BAC16SIM1/2 cell lines were induced with doxycycline and sodium butyrate. The culture medium containing progeny viruses were collected at 72 h post-induction and used to infect HEK293A cells. The progeny virus infectivity was determined by quantification of GFP level by flow cytometry. (TIF) [file ppat.1010504.s004.tif]

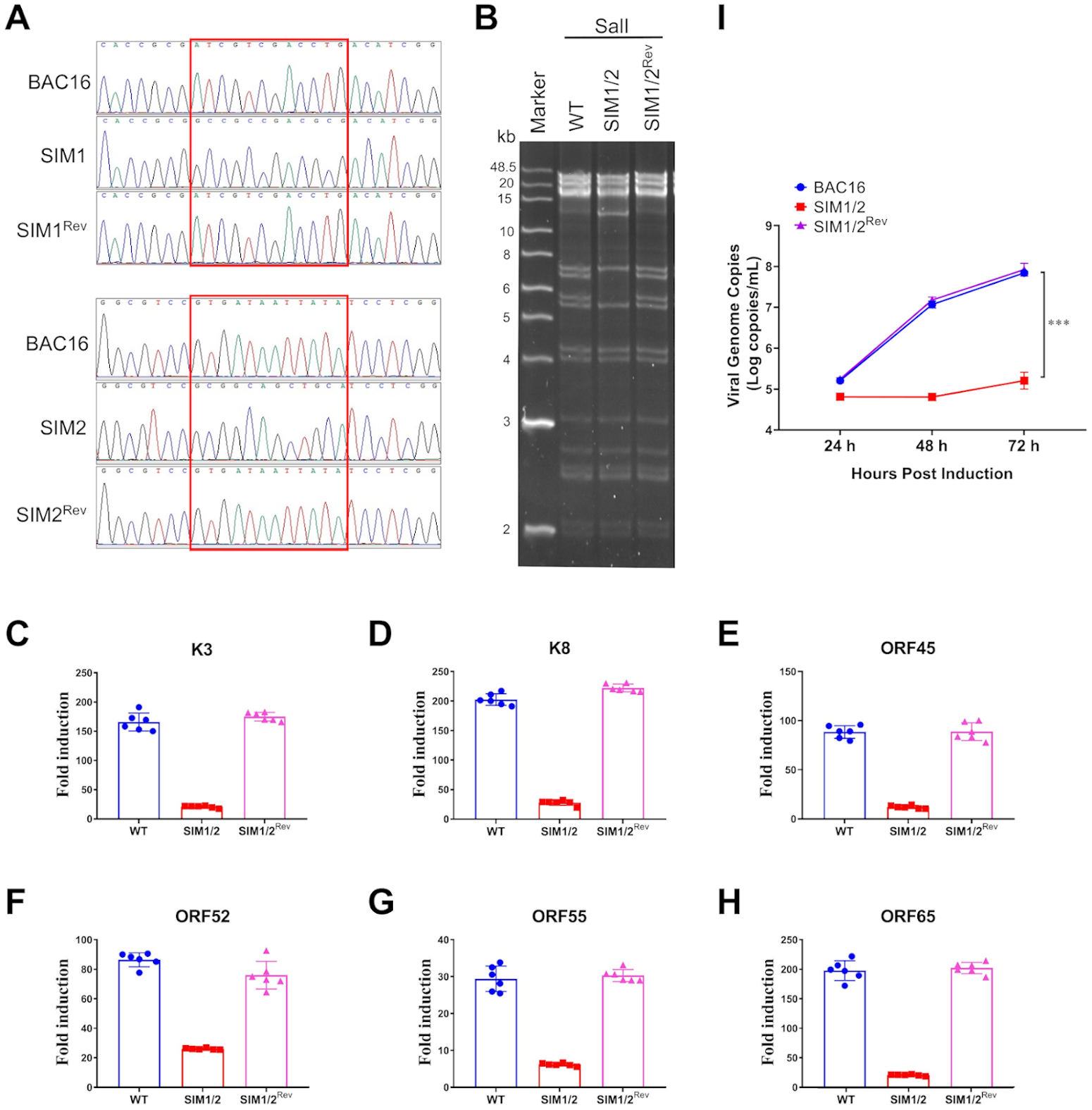

Supplement: S5 Fig — (A-B) Generation of SIM1/2Rev from BAC16SIM1/2 by two-step recombination. The mutated regions were confirmed by DNA sequencing (A) and SalI digestion patterns were determined by agarose gel electrophoresis (B). (C-I) iSLK-BAC16, iSLK-SIM1/2, or iSLK-SIM1/2Rev cells were treated with doxycycline and sodium butyrate to induce lytic replication. At 72 h post-induction, total RNA was extracted and used to evaluate the lytic gene expression by indicated primers (C-H). Total DNA was isolated from culture medium at indicated time point and viral genomic DNA was quantified by qPCR (I). (TIF) [file ppat.1010504.s005.tif]
